# A comparative histological study of the effect of TheraCal LC and biodentine on direct pulp capping in rabbits: an experimental study

**DOI:** 10.1007/s00784-022-04658-9

**Published:** 2022-08-10

**Authors:** Mahmoud Kayad, Azza Koura, Amira El-Nozahy

**Affiliations:** 1 Department of Oral Biology, Faculty of Dentistry, Kafrelsheikh University, Kafr el-Sheikh, Egypt; 2 Department of Oral Biology, Faculty of Dentistry, Alexandria University, Alexandria, Egypt

**Keywords:** Dental pulp capping, TheraCal LC, Biodentine, Tricalcium silicate, Animal model

## Abstract

**Objectives:**

This study histologically compared the effect of TheraCal LC and biodentine on direct pulp capping using a rabbit model.

**Materials and methods:**

A direct pulp capping procedure was performed on 40 mandibular central incisors of 20 healthy, male New Zealand white rabbits. TheraCal LC and biodentine were applied to exposed pulp and 10 randomly selected rabbits were euthanized after the first and second week. Incisors were extracted and prepared for histological processing and examination to check the dentine bridge thickness, continuity, and extent of pulp inflammation. A blinded data analysis was performed, and groups were compared using a Wilcoxon signed-rank test while changes across time within each group were assessed using the Mann–Whitney *U* test.

**Results:**

When comparing the dentine bridge thickness, biodentine showed a significantly thicker dentine bridge in the first and second week (mean 28.16 µm, 33.66 µm), while TheraCal LC showed a dentine bridge in the second week only (mean 15.93 µm). Regarding dentine bridge continuity, biodentine showed a significantly better dentine bridge in the first week. Additionally, there was no difference in the second week. Furthermore, there was no statistically significant difference between each of the materials regarding the extent of inflammation.

**Conclusions:**

Biodentine in vivo showed better results concerning thickness and continuity of the dentine bridge after direct pulp capping in rabbit incisors. Both biodentine and TheraCal LC had a similar inflammatory effect on the pulp.

**Clinical relevance:**

Biodentine is more successful as a direct pulp capping material compared to TheraCal LC.

## Introduction

Vital pulp therapy is a treatment to save and sustain healthy pulp tissue that has been compromised but not destroyed by caries, trauma, or restorative procedures [1]. Vital pulp therapy strategies for permanent teeth include indirect pulp capping, direct pulp capping (DPC), and pulpotomy [2].

DPC is defined as the treatment of exposed vital pulp by sealing pulp wounds with dental material that is placed directly on mechanical or traumatic exposure to facilitate the formation of reparative dentine and the preservation of vital pulp [3].

Various materials from the literature have been proposed for pulp capping such as calcium hydroxide, zinc oxide eugenol cement, bonding agents, glass ionomer cement, Mineral trioxide aggregates (MTA,) and calcium silicate-based materials [4].

MTA is considered a reference material for vital pulp therapy. MTA’s benefits include biocompatibility, bioactivity, sealing ability, and ability to stimulate mineralized tissue development [5–7]. Despite its many advantages, MTA has a few drawbacks including long setting periods, difficulty in handling, and coronal tooth discoloration [5, 8–10].

By improving the downsides of MTA, calcium silicate-based materials have recently emerged and become common due to their similarity to MTA and their applicability in situations where MTA is indicated [11]. While numerous calcium silicate-based products have been recently introduced to the market, two have gained special interest and have become the subject of a wide variety of investigations. Those materials are TheraCal LC (Bisco, Schaumburg, IL, USA) and biodentine (Septodont, Saint Maurdes Fosses, France).

TheraCal LC is a light-curing, resin-modified, calcium silicate-filled single paste. TheraCal LC is composed of Portland cement type III, polyethylene glycol dimethacrylate, bisphenol A-glycidyl methacrylate (Bis-GMA), and barium zirconate [12]. In contrast, Biodentine consists of a powder and a liquid. This powder primarily contains tricalcium silicate and dicalcium silicate, calcium carbonate, and zirconium dioxide, while the liquid is made of calcium chloride [13].

Rabbits, in particular the New Zealand white breed, were used in this research due to their short lifespan, larger tooth size compared to rodent teeth (which is ideal for restorative operations), and pulp tissues equivalent to humans [14, 15]. Also, multiple teeth can be chosen for any experiment in one rabbit and so the number of animals used in one study is decreased. Rabbits have previously been used in dental studies, but few experiments have examined the impact of various pulp capping products on the promotion of pulp wound healing [16, 17].

Previous studies in the literature concerning the histological evaluation of DPC materials on pulp have assessed the pulp response using a Faraco and Holland scoring system [18–23]. This system was modified in this study to achieve more reliable and valid results. Therefore, this study compared TheraCal LC and Biodentine using a modified histological scoring system in a rabbit model.

The null hypothesis of this experimental study is that there is no difference between TheraCal LC and Biodentine regarding dentine bridge thickness, continuity, and extent of inflammation after DPC.

## Materials and methods

This study’s methodology was reported in accordance with ARRIVE (Animal research: Reporting in vivo experiments) guidelines for reporting animal research [24].

### Study aim

This study histologically compared the effect of TheraCal LC and Biodentine as a DPC material on the dental pulp of rabbit mandibular incisors.

### Study design

The study is a comparative animal experiment; rabbits were divided into two groups according to materials used in DPC and these materials were tested at two different time intervals (1 and 2 weeks).

### Study setting

This study was conducted at the Faculty of Dentistry animal house. The histological section of the study was conducted in the Alexandria University histological unit of the Oral Biology Department of the Faculty of Dentistry.

### Sample size

The sample size was estimated assuming 5% alpha error and 80% study power. A pilot study was conducted and according to the obtained data, the mean (SD) dentine thickness after 2 weeks was 17.42 µm (5.4 µm) for the TheraCal LC group and 30.0 µm (11.2 µm) for the biodentine, which resulted in an effect size (d) of 1.43. Based on the difference between the two independent means, the minimum sample size was 9 incisors per group. This increased to 10 incisors to account for processing errors. The total sample size (*N*) = number per group × number of groups × number of time intervals, which results in 10 × 2 × 2 = 40 incisors. Additionally, each tooth was considered an experimental unit. Gpower 3.0.10 was used to calculate the sample results.

### Inclusion and exclusion criteria

Inclusion criteria consisted of healthy, male, New Zealand white rabbits with an approximate weight of 2–2.5 kg and with all mandibular incisors free from caries or fractures. Any rabbit showing systemic illness, wounds, infections, fractures, carious, or periodontally compromised teeth was excluded.

All animals were purchased from the Alexandria University Faculty of Agriculture animal house.

### Randomization and allocation concealment

Each rabbit was provided a number from 1 to 20. Using computer-assisted random number generator software (Prism G. version 5.01. GraphPad Software Inc: San Diego, CA, USA 2007), a random sequence was generated and groups of 10 rabbits were randomly allocated for each interval. Then, for each rabbit, different sides of the mouth were randomly assigned to one material.

The rabbit and material allocation information were recorded and kept in an opaque sealed envelope, which was opened at the time of intervention and euthanasia.

### Experimental procedures

The operation field and rabbits’ incisors were cleaned and disinfected with 0.2% Chlorhexidine and rubber dam isolation was performed under general anesthesia with an intramuscular injection of 35 mg/kg of ketamine HCl and 5 mg/kg of xylazine [25].

Using a high-speed hand piece with a rose head diamond (0.10 ISO standards) and copious water spray, a class V cavity was prepared on the labial surface of the mandibular incisors until a pinkish shadow of the pulp was observed. For every four cavities, a new sterile diamond was used to avoid excessive heating. A sterile bur (0.10 ISO standards) was drilled into the middle of the cavity to expose the pulp in a diameter not exceeding 1 mm without impinging the pulp tissue. Bleeding was controlled using sterile cotton pellets soaked in saline for five minutes.

**Fig. 1:**
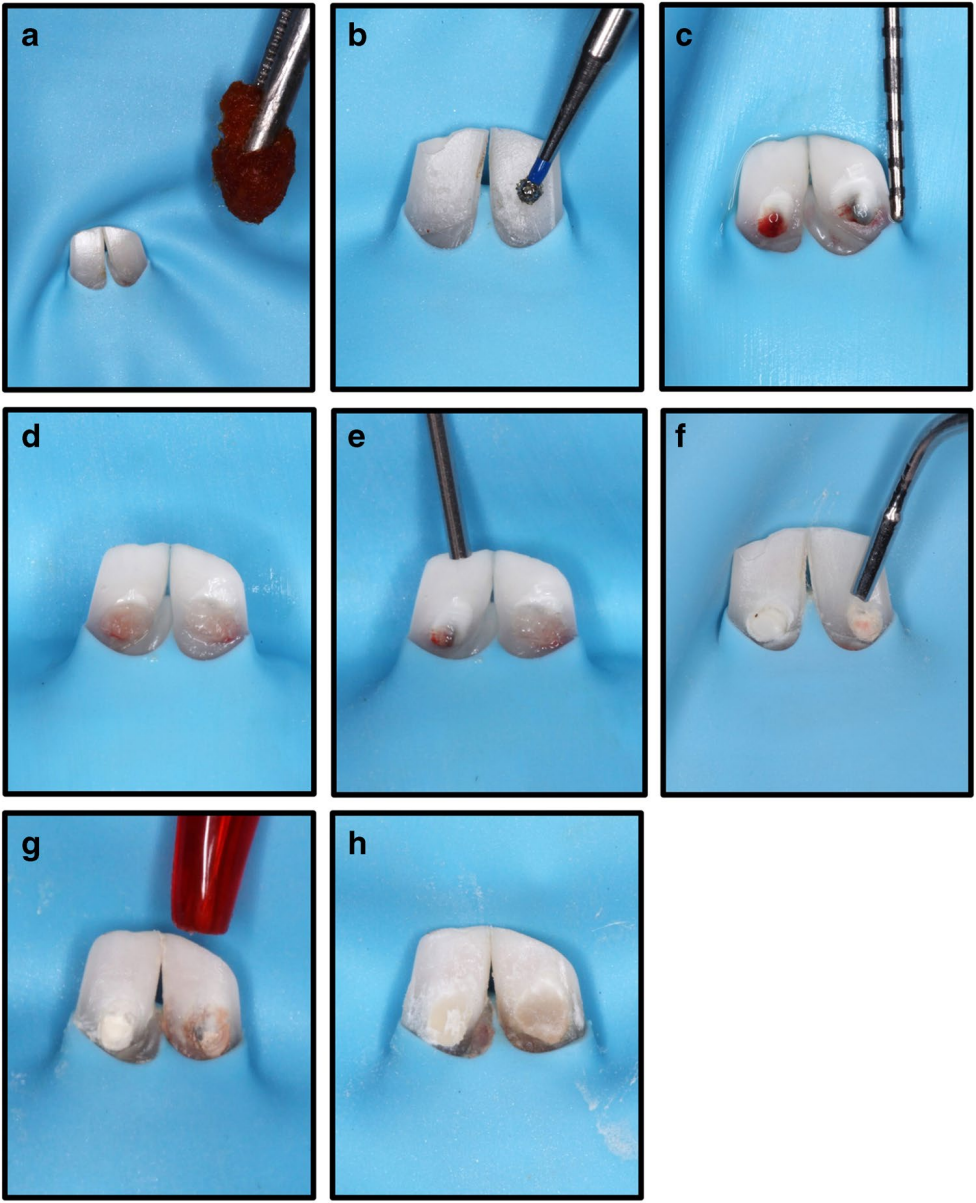
Illustrated steps of direct pulp capping in rabbit mandibular incisors after rubber dam isolation as follows: **A** disinfection using chlorhexidine, **B** the rose head diamond used in the access opening, **C** a class V cavity with central exposure, **D** control of the bleeding using a sterile saline soaked cotton pellet, **E** TheraCal LC application on the right side, **F** biodentine application on the left side, **G** resin-modified restorative glass ionomer application, and **H** finished restorations

Pulp capping materials were mixed according to the manufacturer’s instructions and placed over the exposed pulp without pushing it into the pulp tissue, and materials were assigned according to a predetermined allocation. On both sides, a light cure resin-modified glass ionomer (Riva, SDI, Bauswater, Victoria, Australia) was placed over the capping materials as a final restoration. The steps of this process are shown in Fig. 1.

### Recovery

After the procedure, rabbits were returned to their cages to recover from the anesthesia. Post-operative signs of pain were checked by a veterinarian including an inability to sleep normally, rubbing or scratching at an area, a reluctance to move, a loss of interest in surroundings, and decreased water and food intake. Pharmacologic pain management occurred orally with administration of 1.5 mg/kg meloxicam for 5 days [20].

### Housing and husbandry

Rabbits were individually housed in a stainless-steel cage with wire mesh floors. The temperature, humidity, ventilation, lighting, noise, and chemical and microbiological control were considered by a well-trained veterinarian assistant according to the Canadian Council on Animal Care [25].

### Euthanasia

Ten rabbits were euthanized according to interval allocation by a veterinarian assistant with an intravenous injection of barbiturates at the end of the first and second week [26].

### Histological evaluation

The jaws of the rabbits were removed and fixed in 10% neutral buffered formalin and then decalcified in 20% formic acid buffered with sodium citrate. After decalcification was complete, the teeth were dissected and each specimen was given a code to hide the specimen’s identity (i.e., the material used and time interval).

Then, the specimens were dehydrated, cleaned, and finally infiltrated and mounted in paraffin wax and 5-µ-thick labio-lingual sections from the exposure site were obtained and prepared with hematoxylin and eosin stain [27].

All specimens were included in the analysis, and none were excluded. Representative sections showing whole pulp were examined and evaluated histologically by two different blinded investigators using a light microscope (OPTIKA microscope B-190 TB, Ponteranica, Italy) for extent of the inflammatory reaction and continuity of the dentinal bridge according to a modified scoring system used by Faraco et al. [28] shown in Table 1.

**Table 1:** Scores and criteria for extent of inflammation and continuity of the dentine bridge

Score	Extent of the inflammatory reaction
1	Absent
2	Mild: Inflammation found only in the coronal third of the pulp
3	Moderate: Inflammation extended to the middle third of the pulp
4	Severe: All pulp is inflamed or necrosed
Score	Continuity of the dentinal bridge
1	Complete dentine bridge
2	Little communication of the capping material with dental pulp
3	Only lateral deposition of hard tissue on the walls of the cavity of pulp exposition
4	Absence of a hard tissue bridge and lateral deposition of the hard tissue

The extent of the inflammatory reaction was investigated according to the following criteria [29, 30]:

1. Disturbances in pulp tissue organization and morphology
2. Capillary vasodilatation and engorgement
3. Infiltration of inflammatory cells

Images of representative areas of the complete dentinal bridge were captured using a mounted and calibrated digital camera (Canon 1300D, Canon Inc., Tokyo, Japan) at × 40 and × 100 magnifications and then gauged at three different points (thinnest, thickest, and midmost) by a blinded investigator using the Image J program (version 1.80_172, National Institutes of Health, Bethesda, MD, USA).

### Blinding

For each animal, three different investigators were involved as follows: the first investigator (M.K.) applied both materials and was blinded to rabbits’ interval allocation. However, further blinding was impossible until euthanasia occurred due to the different formfactor/application method of both materials.

The second (A.K.) and third (A.N.) investigators were blinded to the allocation of rabbits and materials and evaluated both dentine bridge continuity and the extent of inflammation. The third investigator (A.N.) gauged the dentine bridge thickness.

The first investigator (M.K.) did not participate in the histological evaluation or histomorphometric analysis to avoid conscious or unconscious bias or any other factor that was irrelevant to the pulp response based on the material used.

### Reliability

Inter- and intra-examiner reliability was assessed by repeating the evaluation and measurements approximately 6 to 8 h apart by the same investigator.

### Statistical analysis

A blind data analysis was performed; normality was checked using the Shapiro–Wilk test, box plots, and descriptive statistics and was not normally distributed. The dentine thickness and inflammation scores were presented using mean, median, standard deviation, and inter quartile range (IQR) while the continuity of the dentine bridge was presented using a counting process and percentage. Groups were compared using the Wilcoxon signed-rank test, while changes across time within each group were assessed using the Mann–Whitney *U* test. The distribution of scores was compared using a Pearson chi-square test. The reliability of dentine thickness was assessed using an intraclass correlation coefficient (ICC) and Cohen’s Kappa statistics for inflammation scores and continuity of the dentine bridge. The significance level was set at a *p*-value of 0.05 and all tests were two tailed. Data analyses were conducted using SPSS for Windows version 23.

## Results

### Tolerance of experimental procedures

Not all animals showed obvious signs of systemic illness during the study period. Post-anesthetic drowsiness and a lack of appetite transiently occurred during the first 2 days after the DPC procedure. After this period, eating returned to normal, which indicated that the restorations did not cause interference. Additionally, restorations were regularly examined during treatment and remained stable until the end of treatment.

### Reliability

Intra-examiner reliability for dentine thickness (ICC) was 0.90 and inter-examiner reliability using Kappa values for the inflammatory reaction and the continuity of the dentinal bridge ranged from 0.85 to 0.90.

### Thickness and continuity of the dentine bridge (Tables 2 and 3)

**Table 2:** Comparison of dentine thickness between the TheraCal LC group and the biodentine group at two time points

		TheraCal LC (*n* = 10)	Biodentine (*n* = 10)	Mann–Whitney Test (*p*-value)
1st week	Mean (SD)	0	28.16 (13.34)	4.038 (< 0.0001*)
	Median (IQR)	0	27.84 (24.32)	
	Min–Max	0	9.54–47.30	
2nd week	Mean (SD)	15.93 (15.16)	33.66 (15.77)	2.050 (0.043*)
	Median (IQR)	13.38 (25.88)	24.46 (21.76)	
	Min–Max	0–47.67	17.36–66.90	
Wilcoxon signed-rank test (*P*-value)		2.366 (0.018*)	0.459 (0.646)	

*Statistically significant difference at *p* ≤ 0.05

**Table 3:** Comparison of the dentinal bridge continuity between the TheraCal LC group and the biodentine group at two time points. ^*^Statistically significant difference at *p* ≤ 0.05

		TheraCal LC (*n* = 10)	Biodentine (*n* = 10)	Mann–Whitney test (*p*-value)
1st week	Median (IQR)	2.0 (1.0)	1.0 (1.0)	4.147 (< 0.0001*)
	Min–Max	2.0–3.0	1.0–1.0	
2nd week	Median (IQR)	1.0 (1.0)	1.0 (1.0)	0.067 (0.280)
	Min–Max	1.0 – 2.0	1.0–1.0	
Wilcoxon signed-rank test (*p*-value)		2.640 (0.008*)	0.000 (1.00)	

In the first week, a complete dentine bridge did not form in the TheraCal LC group, and seven samples showed a score = 2 while three samples showed a score = 3. However, in the Biodentine group, all samples showed complete dentine bridge formation (score = 1) with average thickness = 28.16 µm.

**Fig. 2:**
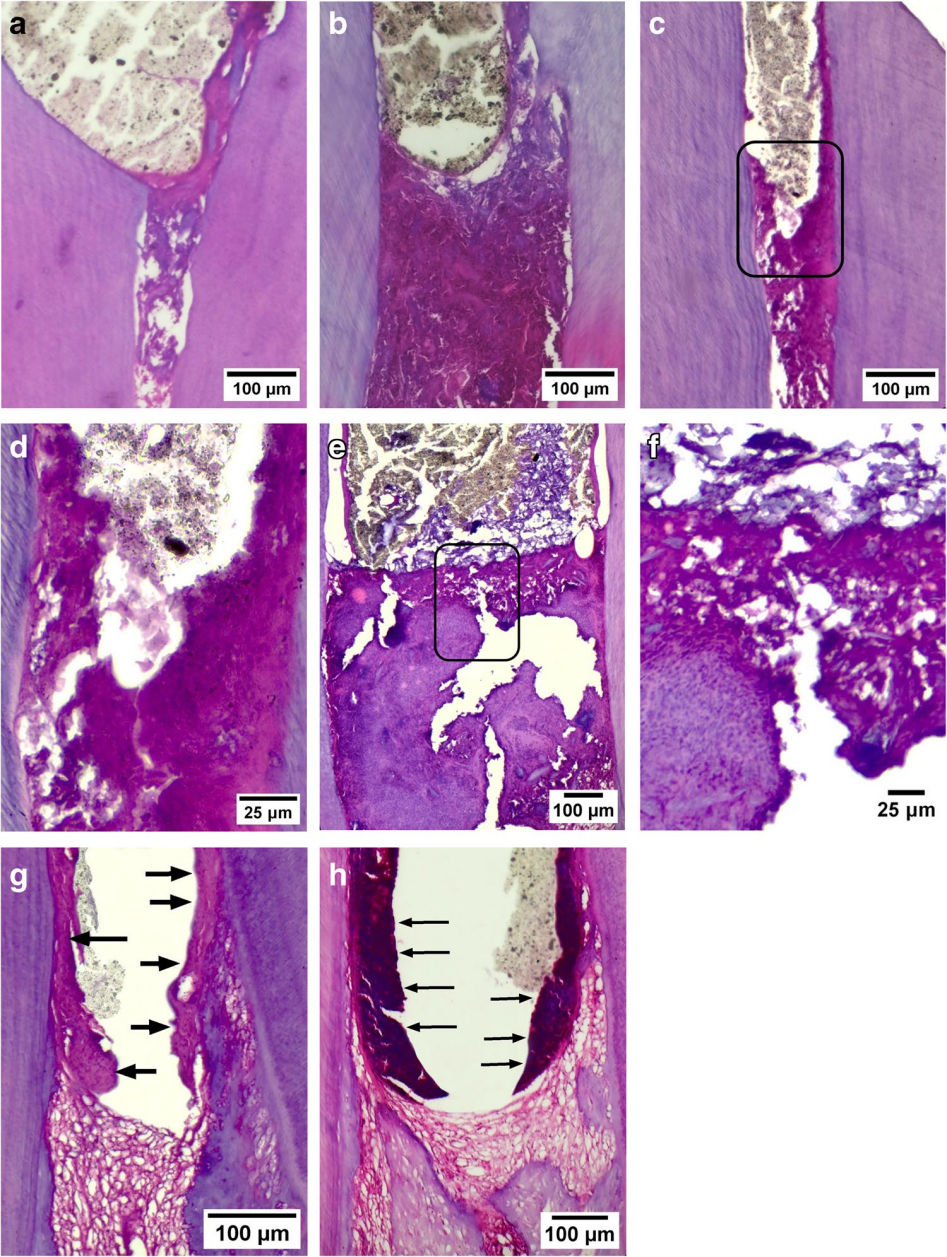
Decalcified sections that were hematoxylin–eosin stained. **A**, **B**, **C**, **E**, **G**,** H** × 100 magnification, **D**,** F** × 200 magnification. **A**, **B** Complete dentine bridge of different thickness (score = 1). **C**, **E** Communication of the capping material with the dental pulp (score = 2) higher magnification of previous inset is also shown in **D**,** F**. **G**, **H**. Formation of new hard tissue only on the lateral walls (arrows) (score = 3)

In the second week, seven TheraCal LC samples showed a score = 1 with average thickness = 15.93 µm while three samples had a score = 2. However, all samples of Biodentine showed complete dentine bridge formation (score = 1) with an average thickness = 33.66 µm (Fig. 2).

### Extent of the inflammatory reaction (Table 4)

**Table 4:** Comparison of inflammation scores between the TheraCal LC group and biodentine group at two time points

		TheraCal LC (*n* = 10)	Biodentine (*n* = 10)	Mann–Whitney test *(p*-value)
1^st^ week	Median (IQR)	4.0 (1.0)	3.0 (1.0)	0.872 (0.481)
	Min–Max	3.0–4.0	3.0–4.0	
2^nd^ week	Median (IQR)	3.0 (2.0)	3.0 (0.25)	0.168 (0.912)
	Min–Max	2.0–4.0	3.0–4.0	
Wilcoxon signed-rank test (p-value)		1.299 (0.194)	0.816 (0.414)	

In the first week, four TheraCal LC samples showed a score = 3 and six samples showed a score = 4. In the biodentine, six samples showed a score = 3 and four samples showed a score = 4.

**Fig. 3:**
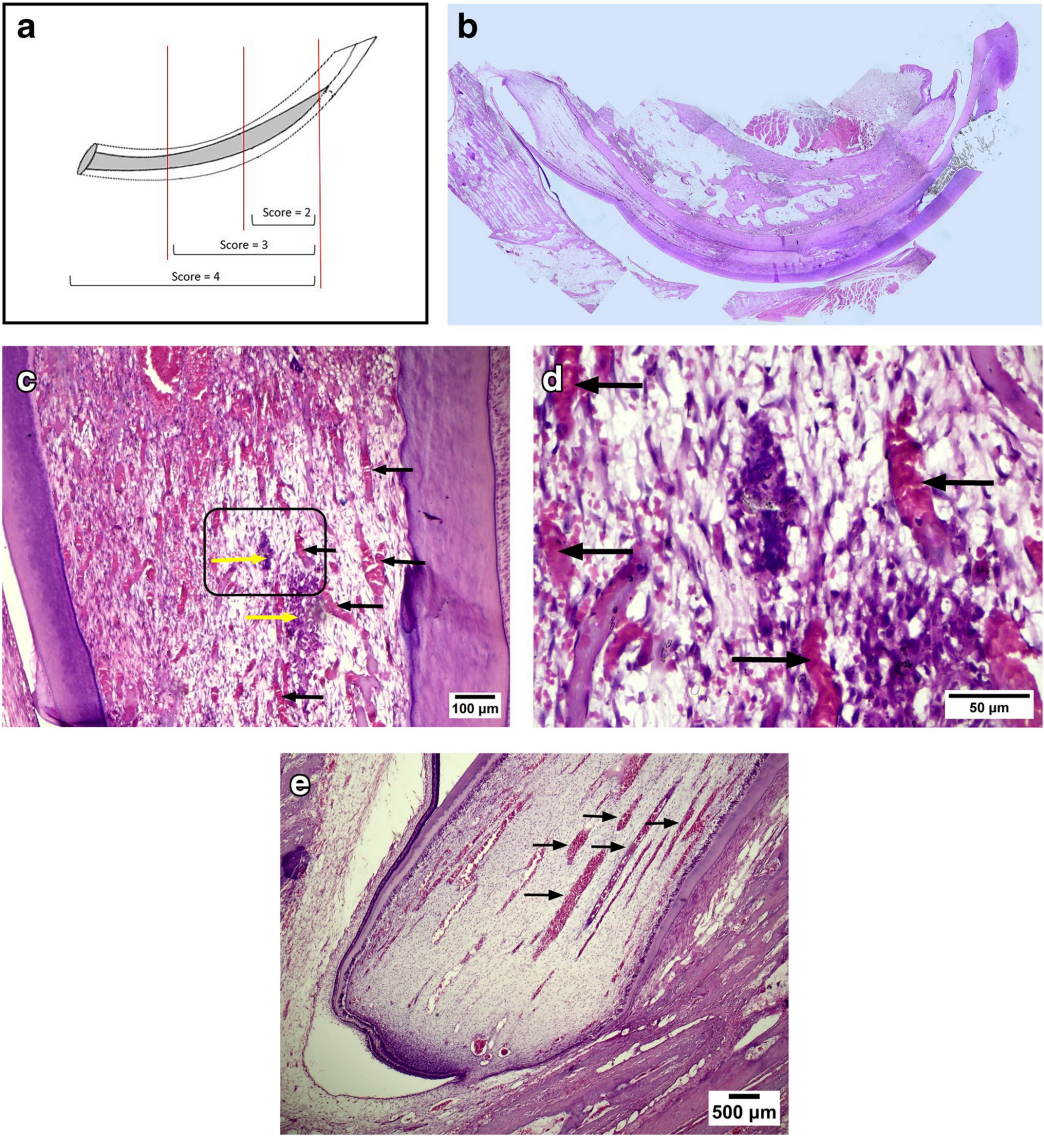
**A** Diagram of mandibular incisor illustrating extent of inflammation scoring. **B** Computer enhanced low power decalcified longitudinal section for mandibular rabbit incisor. **C**, **D**, **E** *D*ecalcified sections that were hematoxylin–eosin stained. **C** × 100 magnification of pulp middle third showing pulp disorganization in upper left corner, wide engorged blood vessels (black arrows). ***D*** Higher magnification × 400 of previous inset showing infiltrated inflammatory cells (yellow arrows). ***E*** × 40 magnification showing wide engorged blood vessels located in apical third of the pulp (black arrows)

Meanwhile, in the second week, three TheraCal LC samples showed a score = 2, three samples showed a score = 3, and four samples showed a score = 4, whereas eight biodentine samples showed a score = 3 and two samples showed a score = 4 (Fig. 3).

## Discussion

This study assessed the pulpal response to TheraCal LC and Biodentine after DPC in a rabbit model. Generally, animal experiments are important as they yield results that generate useful knowledge that supports an understanding of a range of biological mechanisms, and human results can be hypothesized using this information.

This study focused more on histological results than long-term clinical outcomes. Dentine bridge thickness, continuity, and extent of the pulp inflammation after DPC are the most important histological features for predicting future clinical outcomes. In our study, a modified scoring system was used to obtain valid and reliable results.

Faraco et al. evaluated dental pulp after DPC and found that a hard tissue bridge formed in dog teeth based on a scoring system according to the criteria described in Table 5. Each histomorphological event was graded on a scale of 1 to 4 with 1 the best and 4 being the worst.

**Table 5:** Scoring system

SCORE	Hard tissue bridge continuity
1	Complete
2	Little communication of the capping material with dental pulp
3	Only lateral deposition of hard tissue on the walls of the cavity of pulp exposition
4	An absence of the hard tissue bridge and lateral deposition of hard tissue
Thickness (Evaluated with a micrometric ocular in three different points of the bridge)	
1	Up to 250 µm
2	From 150 to 249 µm
3	From 1 to 149 µm
4	Partial or absent bridge
Intensity of the inflammatory reaction (acute and chronic processes) (evaluated in various places with a magnification of 400 ×)	
1	Absent or very few cells
2	Mild: an average number of less than 10 cells
3	Moderate: an average number 10–25 cells
4	Severe: an average number greater than 25 cells

In our study, we noted that the dentine bridge thickness scoring system had low validity due to physiologic differences as it was developed for dogs’ teeth and at specific time intervals. However, considering the intensity of inflammation, it was also observed that counting inflammatory cells was not a reliable method and was replaced by an easily detectable criteria using eosin and hematoxylin stain.

Generally, biodentine showed better results regarding the dentine bridge thickness and continuity. Additionally, there was no statistically significant difference regarding pulp inflammation.

Based on previous studies, TheraCal LC showed properties of calcium release [31–34]. The bioavailability of calcium ions plays a crucial role in the material-induced development and differentiation of human dental pulp cells and the creation of new mineralized hard tissues. Overall, TheraCal LC produces calcium but at a lower level than biodentine [31]. This may better explain the dentine bridge formation for Biodentine in this study. This is consistent with a recent randomized controlled clinical trial conducted by Peskersoy C et al. [35].

Regarding pulp irritation prevention and vitality preservation, the cytotoxicity and biocompatibility of the pulp capping material is highly important. TheraCal LC and Biodentine interactions with dental pulp were investigated and showed that TheraCal LC has a greater inflammatory activity and lower bioactive capability than biodentine [35]. Although biodentine showed lower inflammation scores, there was no statistically significant difference between either material, this could be explained by the rapid setting of TheraCal LC via light polymerization and improved mechanical sealing achieved during procedures. Furthermore, the lower-than-expected alkaline pH of Biodentine just after setting may cause pulpal inflammation [35].

When comparing both intervals of the same materials, TheraCal LC showed better results in the second week regarding continuity and the thickness of the dentine bridge, which can be explained by the reduction in hydroxyl ion release after 7 days. This reaches a physiological pH that may create a favorable environment for pulp cell viability and metabolic activity during the formation of new/reparative tertiary dentine [33]. Considering pulp inflammation scores, TheraCal LC showed lower pulp inflammation scores in the second week, which can be explained (as mentioned previously) but was non-significant in our study. Meanwhile, Biodentine showed no significant difference between both intervals regarding the continuity and thickness of the dentine bridge and pulp inflammation scores. A possible justification for this is the constant hydroxyl ion release of Biodentine over a 28 day period [36].

The use of rabbits in DPC investigations is uncommon, and other animals more commonly used are dogs, cats, pigs, and monkeys [21, 37–39]. However, rabbits are surpassed by their short lifespan and economical aspects regarding purchasing and maintenance. Also, they are characterized by their suitable teeth size, which can be used to test restorative operations. Although rabbits’ incisors are characterized by continuous eruption and incisal attrition, results obtained from testing DPC materials on their pulp coincides with multiple in vivo and in vitro studies [33, 35, 36, 38, 40], which may indicate that rabbit incisors are legible for testing new DPC materials over short time intervals.

However, multiple difficulties need to be addressed as follows: the access opening in some cases due to pulp recession, histological sectioning due to the curved nature of rabbit incisors, and an inability to increase time intervals more than two weeks due to continuous eruption and incisal attrition, which may lead to a loss of the restoration under investigation.

This study compared the pulpal response of TheraCal LC and Biodentine after DPC in an animal model. However, this study has some limitations because it was performed in a noninfectious model, which does not accurately reflect the clinical situation of inflammation that is present in the pulp. Additional validation through infection models is required. Also, materials were investigated for short intervals that were limited by the selected animal model.

## Conclusions

In conclusion, this study provides a new and reliable method for comparing the effect of various materials on dental pulp, which can be applied to different animal models and at different intervals.

This in vivo study found that the inflammatory pulpal responses to TheraCal LC and Biodentine after DPC were the same. However, biodentine showed better results regarding the dentine bridge thickness and continuity.

## Data Availability

All data generated or analyzed during this study are included in this published article and its supplementary information files.

## Abbreviations

DPC: Direct pulp capping
MTA: Mineral trioxide aggregates
Bis-GMA: Bisphenol A-glycidyl methacrylate
